# Reduction of rupture risk in ICA aneurysms by endovascular techniques of coiling and stent: numerical study

**DOI:** 10.1038/s41598-023-34228-2

**Published:** 2023-05-03

**Authors:** Ali Rostamian, Keivan Fallah, Yasser Rostamiyan

**Affiliations:** Department of Mechanical Engineering, Sari Branch, Islamic Azad University, Sari, Iran

**Keywords:** Biomedical engineering, Mechanical engineering

## Abstract

The initiation, growth, and rupture of cerebral aneurysms are directly associated with Hemodynamic factors. This report tries to disclose effects of endovascular technique (coiling and stenting) on the quantitative intra-aneurysmal hemodynamic and the rupture of cerebral aneurysms. In this paper, Computational Fluid Dynamic are done to investigate and compare blood hemodynamic inside aneurysm under effects of deformation (due to stent) and coiling of aneurysm. The blood stream inside the sac of aneurysm as well as pressure and OSI distribution on the aneurysm wall are compared in nine cases and results of two distinctive cases are compared and reported. Obtained results specifies that the mean WSS is reduced up to 20% via coiling of the aneurysm while the deformation of the aneurysm (applying stent) could reduce the mean WSS up to 71%. In addition, comparison of the blood hemodynamic shows that the blood bifurcation occurs in the dome of aneurysm when endovascular technique for the treatment is not applied. It is found that the bifurcation occurs at ostium section when ICA aneurysm is deformed by the application of stent. The impacts of coiling are mainly limited since the blood flow entrance is not limited in this technique and WSS is not reduced substantial. However, usage of stent deforms the aneurysm angle with the orientation of parent vessel and this reduces blood velocity at entrance of the ostium and consequently, WSS is decreased when deformation of the aneurysm fully occurs. These qualitative procedures provide a preliminary idea for more profound quantitative examination intended for assigning aneurysm risk of upcoming rupture.

## Introduction

The precise evaluation of the endovascular techniques is crucial for the selection of efficient treatment for saccular aneurysms^1–4^. Although risk of rupture is moderately low in unruptured cerebral aneurysms, protective interventions are frequently considered owing to the poor diagnosis of intracranial haemorrhage^5–8^. Existing treatments of intracranial aneurysms convey a small but momentous risk that could surpass the natural risk of aneurysm rupture, and this augments the importance of performance of different endovascular techniques for reduction of the rupture risk of cerebral aneurysms for clinicians^9–14^. Pre-interventional analysis of the aneurysm is mainly related to size of aneurysm in which rupture risk of larger aneurysm is higher than lower ones^15–19^. Although this logic seems reasonable, some reports indicates rupture of small aneurysm is not ignorable. Hence, the importance of hemodynamic analysis and related factors for the evaluation of the aneurysm rupture is raised^20–23^. Previous reports and articles confirmed that the mechanisms of aneurysm growth and rupture are related to hemodynamic factors^24–29^. Besides, hemodynamic factors also present valuable measure for the estimation of aneurysm rupture. A few geometric measures, for example shape descriptors and aspect ratio, are considered in former studies as substitutes for hemodynamic information^30–34^. Although these factors could be suitable for refining risk evaluation, the underlying mechanisms are difficult to connect rarely disclosed via these values^35–38^.

These methods have their own cons and pros and comparison of these techniques are seldom reported for ICA aneurysms^39–44^. This study tries to investigate the effects of these two techniques on hemodynamic factors. CFD technique is used for modeling of the pulsatile blood flow and achieve hemodynamic factors (i.e. OSI and WSS) for estimation of the aneurysm rupture.

## Materials and methods

It is confirming that all methods were carried out in accordance with relevant guidelines and regulations. Besides, all experimental protocols were approved by of the Ca' Granda Niguarda Hospital and it is confirmed that informed consent was obtained from all subjects and/or their legal guardian(s). All study are approved by Ca' Granda Niguarda Hospital ethics committee^45^.

Nine different patients with intracranial aneurysms are investigated in present study. Due to similarity between the hemodynamic of some of these aneurysms, results of two most distinctive models are presented in this paper. For the selection of the aneurysm, the chosen aneurysms have high angles with parent vessel orientation. Besides, the sac Neck Vessel angle is almost identical in these two chosen models. Selected cases of 34 and 06 are related to women and man, respectively. Hence, HCT value of the female and male cases are 0.4 and 0.45, respectively. Details of chosen aneurysms are presented in Table 1.

**Table 1:** Details of selected aneurysms.

Case ID	Sac ostium section area (mm^2^)	Sac neck vessel angle (°)	Sex
06	34.9	31.4	Male (HCT = 0.45)
34	41.3	39.8	Female (HCT = 0.40)

The geometry (.stl) of selected aneurysm is obtained from Aneurisk Website^45^ which offer full shape of the ICA aneurysm with case number of CO34 and C006. The surface of chosen aneurysm is produced by ICEM software. Then, sac section is split for the implementation of the porous as coiling technique. Deformation is also applied according to angle of the parent vessel angle with normal vector of ostium surface. The main concept for the deformation is to preserve the straight angle of the main parent vessel to reduce the blood entrance in to sac section area. Details of ICA ostium and neck vessel angles are presented in Table 1.

In this work, Navier–Stokes equations are solved for computational modeling of the blood stream. It is assumed that the blood stream is non-Newtonian, incompressible and transient^46–48^. One-way FSI model is used for the modeling the blood interactions with aneurysm wall. Casson model is developed for the calculation of the viscosity term in the main governing equations. For modeling of coiling, sac section is assumed to fill with porous material with specific details associated with size of aneurysm^49–55^. Applied porosity for the chosen aneurysms are presented in Table 2.

**Table 2:** Details of applied porosity.

Porosity	Permeability (m^2^)	Viscous resistance (m^−2^)
C0006		
0.791	1.2555e−7	7,964,902
0.545	4.1065e−8	24,351,202
C0034		
0.791	2.9310e−7	3,411,832
0.545	9.5868e−8	10,431,042

**Figure 1:**
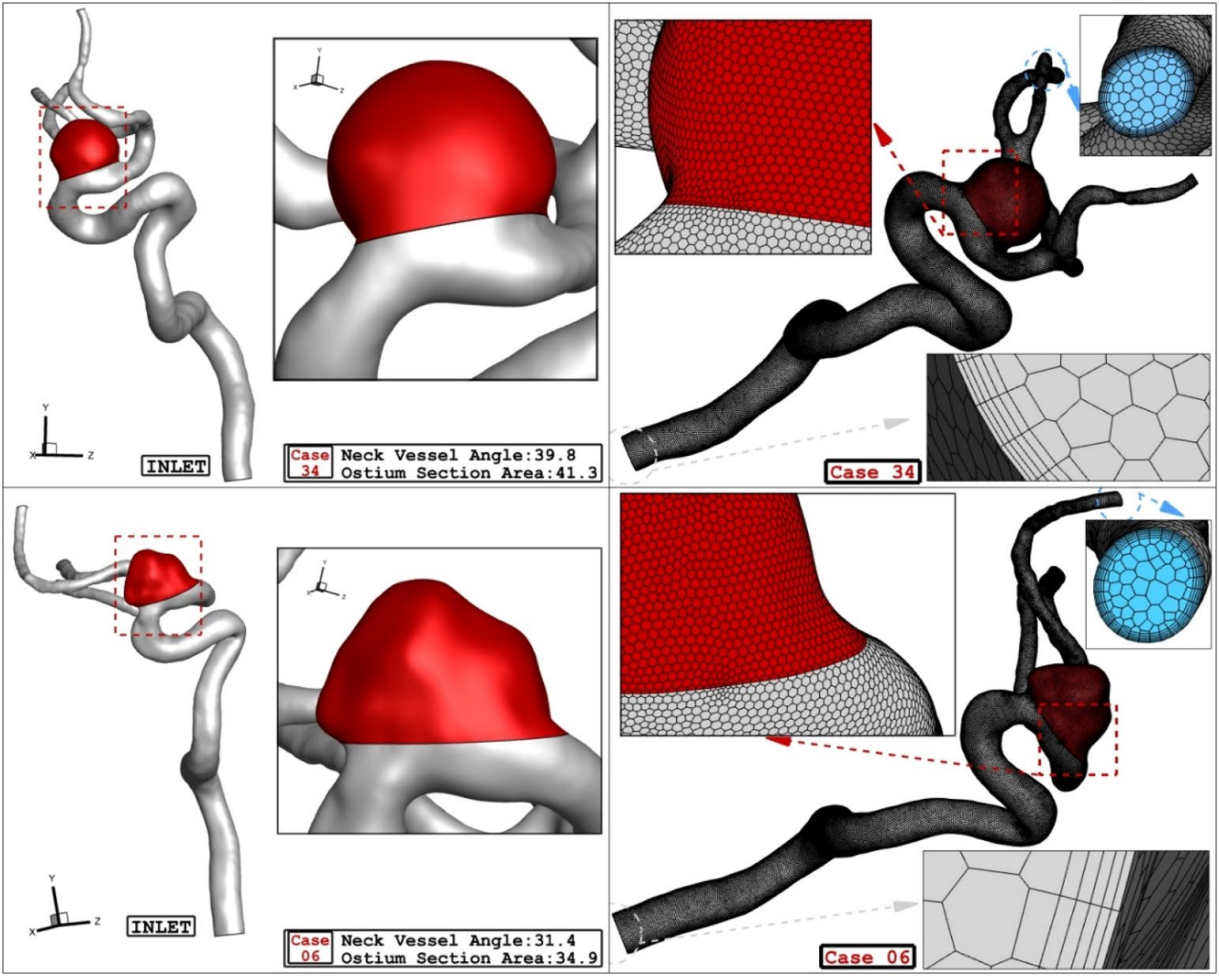
ICA aneurysm and grid generation of 2 different cases.

**Figure 2:**
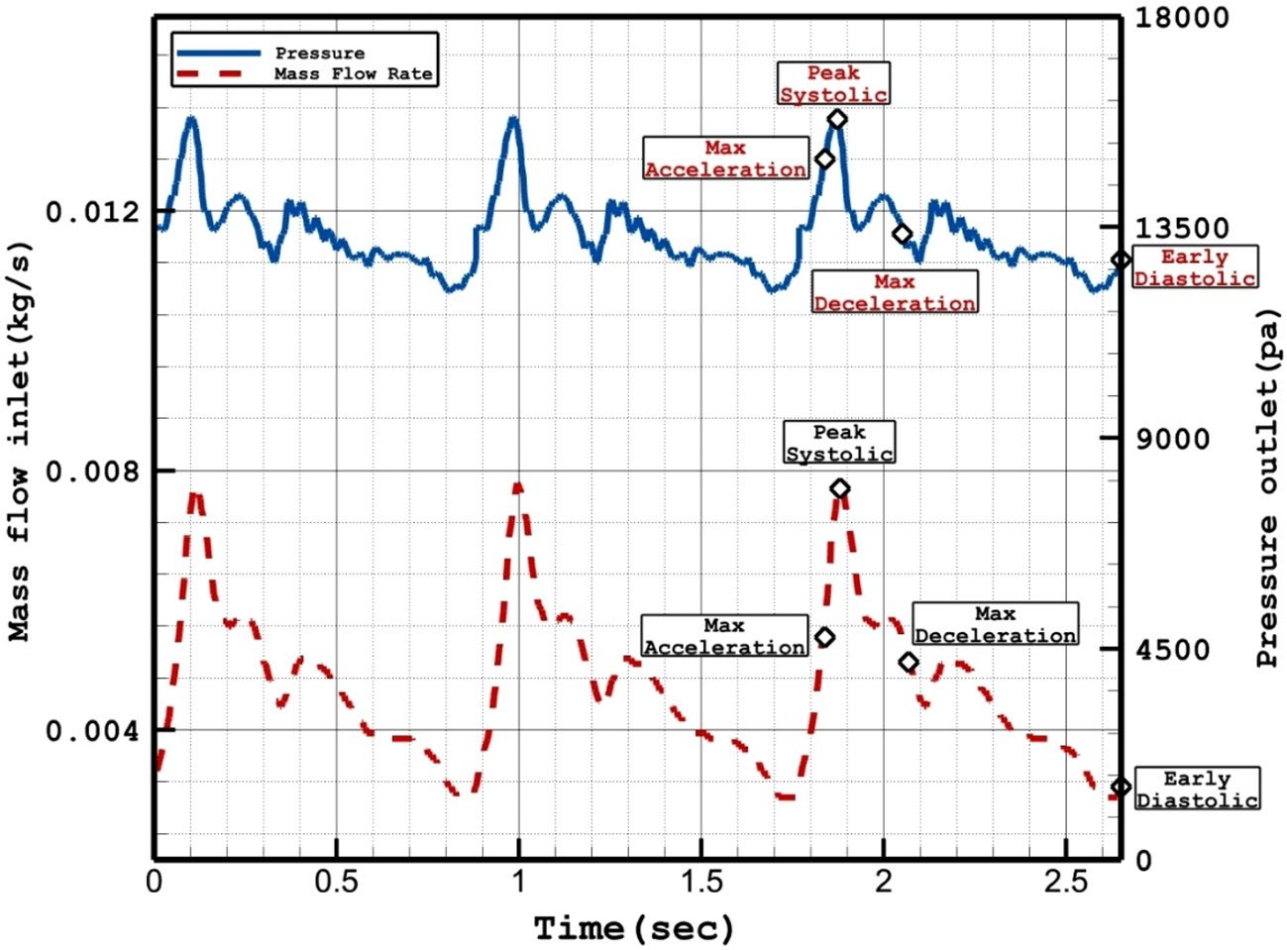
Applied mass and pressure profile at inlet and outlets.

Figure 1 illustrates the produced grid for the chosen aneurysms. Boundary layer is used for the grid production near the vessel wall as depicted in Fig. 1. Since the blood flow has pulsatile pattern, the mass flow profile of Fig. 2 is applied at inlet and equivalent pressure profile present in Fig. 2 is used for outlet pressure^29,31^. There are four distinctive stage in the figure for the comparison of the results. To ensure about the archived data, results of 3rd blood cycle is presented in this work and reported OSI value is for last time step (step = 3000). Maximum blood velocity occurs in the peak systolic and this condition is evaluated as wore case scenario. This study tried to investigate the rheology aspects of blood stream^56–60^.

## Results and discussion

### Coiling effects

**Figure 3:**
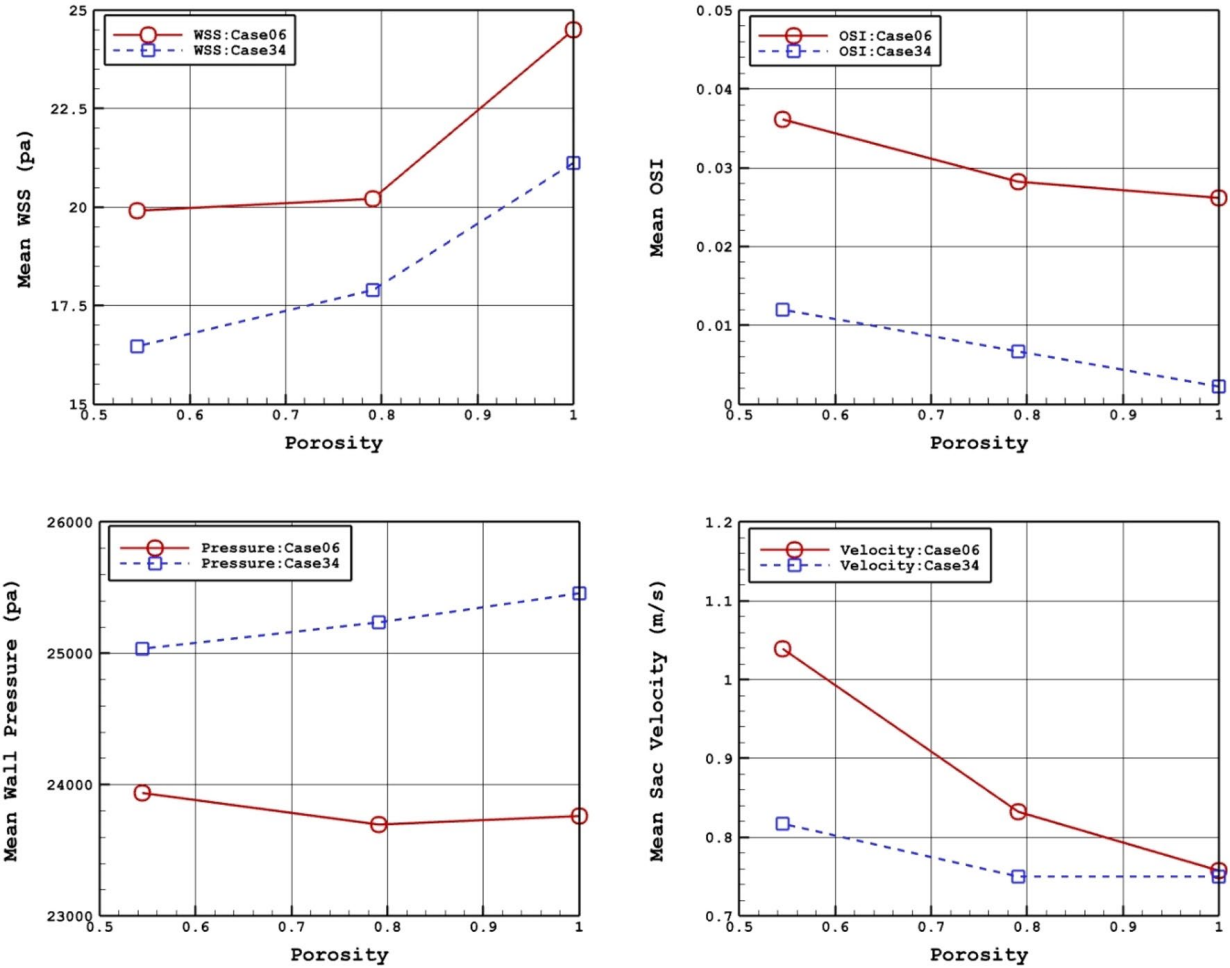
Porosity effects on mean values of WSS, OSI, sac pressure, and sac velocity.

To investigate the impacts of coiling, Table 3 compares hemodynamic characteristics of the blood stream for selected cases in two porosities of 0.791 and 0.545 with a simple model. Mean WSS, OSI, wall pressure and velocity are presented in this table. For better evaluation of the obtained results, Fig. 3 plots the variation of these factors. Our records show that increasing the porosity almost raise the mean WSS while mean sac velocity and OSI value is decreased. However, no specific trend is observed in mean wall pressure.

**Table 3:** details of obtained hemodynamic results.

Porosity	WSS_mean (Pa)	OSI_mean	Wall pressure_mean (Pa)	Aneurysm velocity_mean (m/s)
C0006				
Without coiling	24.5	0.026	23,760	0.76
0.791	20.0	0.028	23,699	0.83
0.545	19.9	0.036	23,939	1.04
C0034				
Without coiling	21.1	0.002	25,456	0.75
0.791	17.9	0.007	25,236	0.75
0.545	16.5	0.012	25,035	0.82

**Figure 4:**
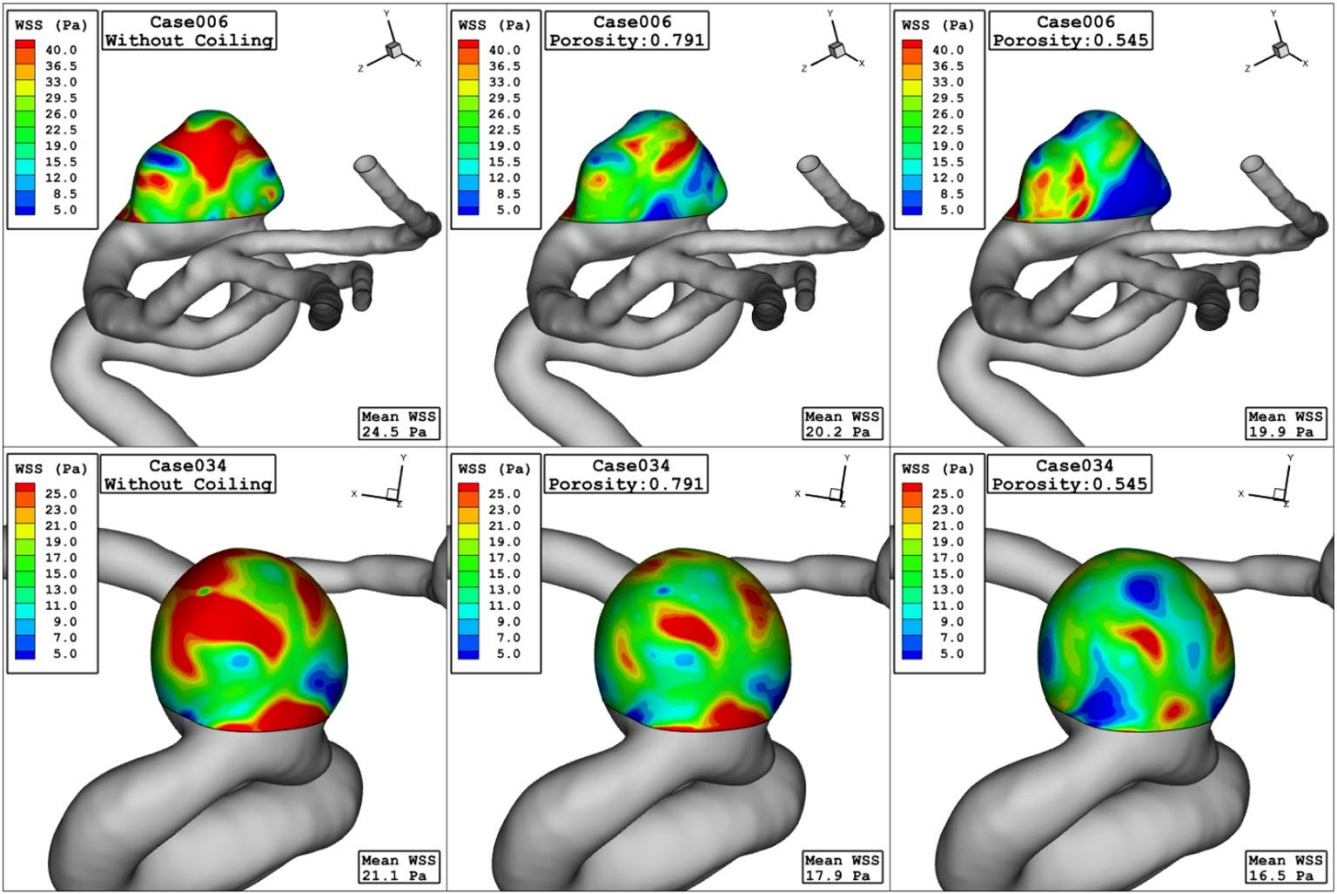
WSS contours (peak systolic) in different porosity.

**Figure 5:**
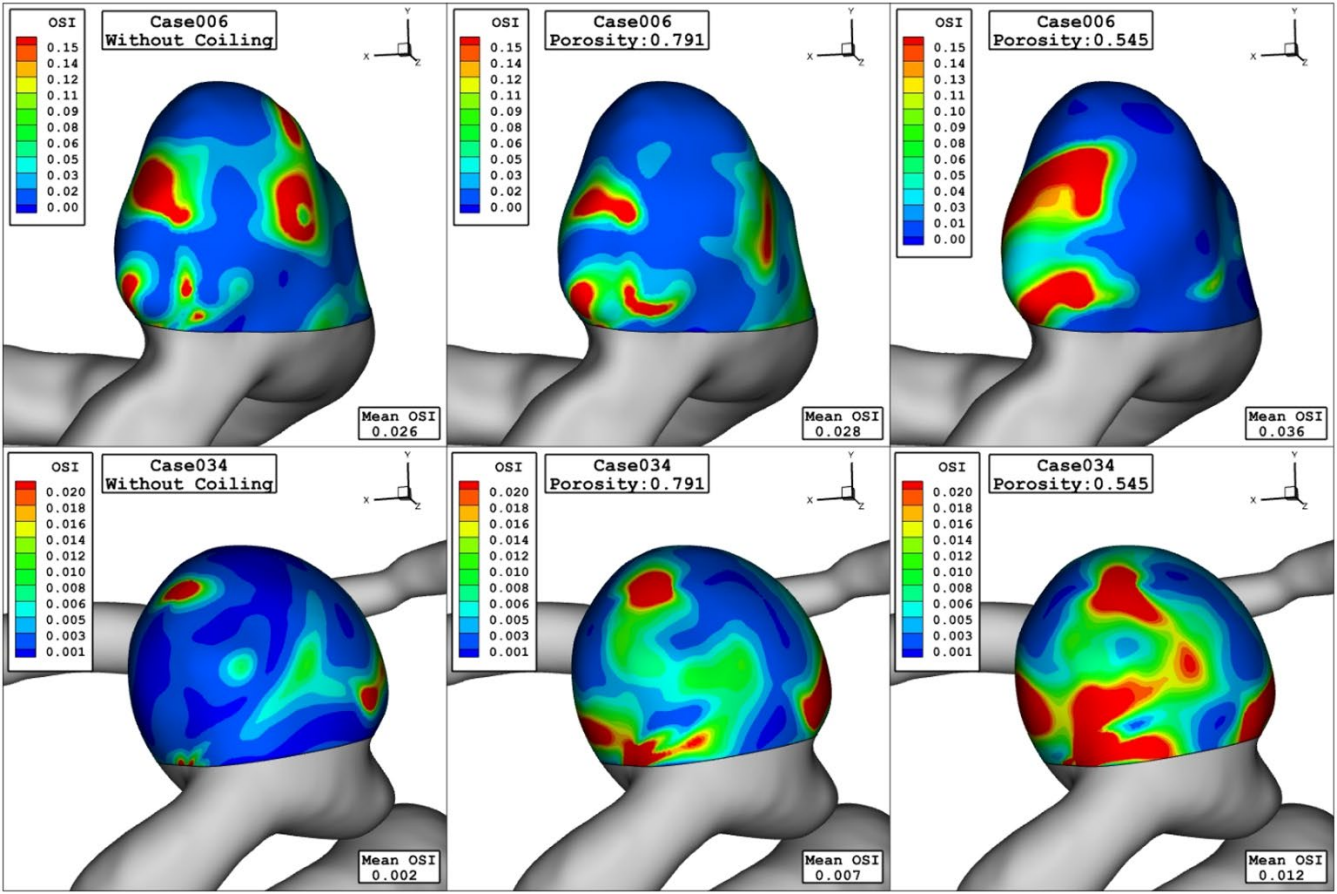
OSI contours (early diastolic) in different porosity.

Comparison of the WSS in the sac of the aneurysm at peak systolic stage are demonstrated in Fig. 4 for different porosity values. The value of the WSS for Case006 is higher than that of Case034. By decreasing of the porosity, permeability of the domain increases and less interactions of the blood occurs with the aneurysm wall. Thus, the high WSS region on sac surface is limited. Figure 5 displays distribution of the OSI on the aneurysm wall for different porosity values at early diastolic stage. In case006, high OSI region transfers to the neck of aneurysm by the decreasing of the porosity values. However, high OSI region extends in the case034 by decreasing the porosity.

**Figure 6:**
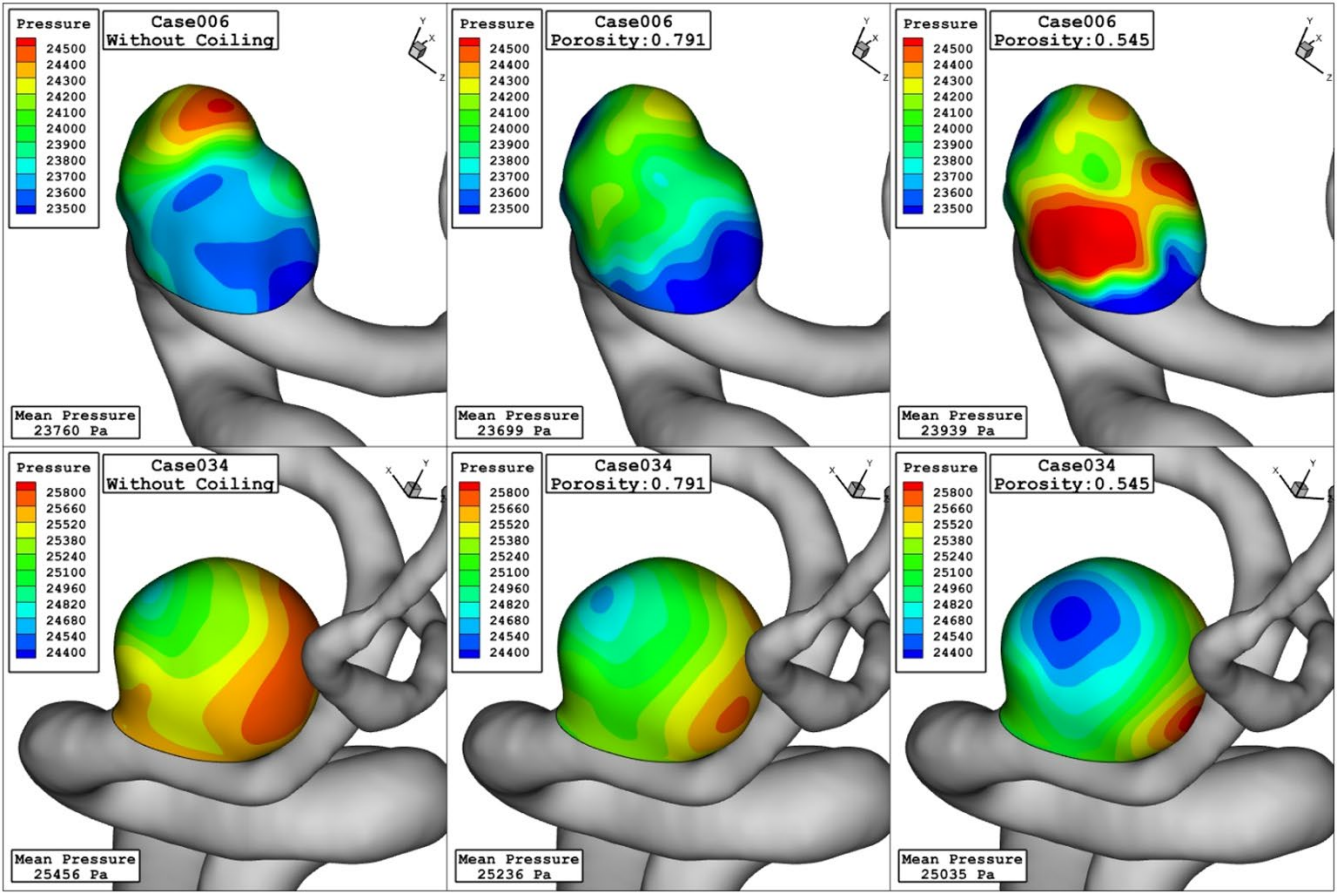
Pressure contours (peak systolic) in different porosity.

**Figure 7:**
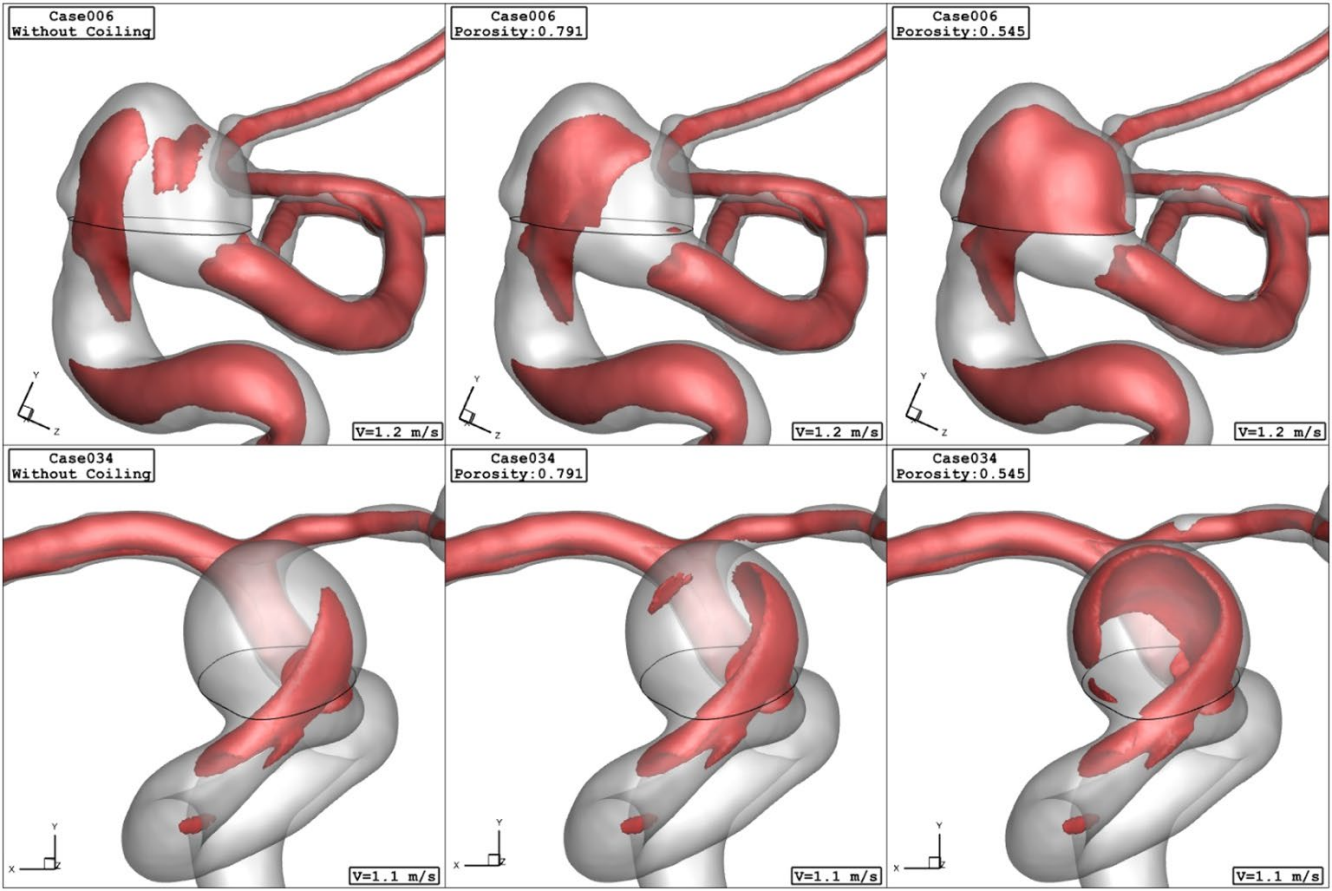
Iso-surface (velocity at peak systolic) in different porosity.

Figure 6 displays the domain with high pressure and how pressure distribution alters by changing the porosity. Decreasing the porosity values declines pressure on the dome of aneurysm surface in the case006. The feature of the blood flow inside the aneurysm is displayed in Fig. 7. Effects of the coiling on blood hemodynamic inside these two case are not identical. The feature of iso-surface velocity (v = 1.2 m/s) indicates that in the high-velocity blood stream tends to move near the wall by decreasing the porosity at peak systolic stage.

### Stent-induced deformation

**Figure 8:**
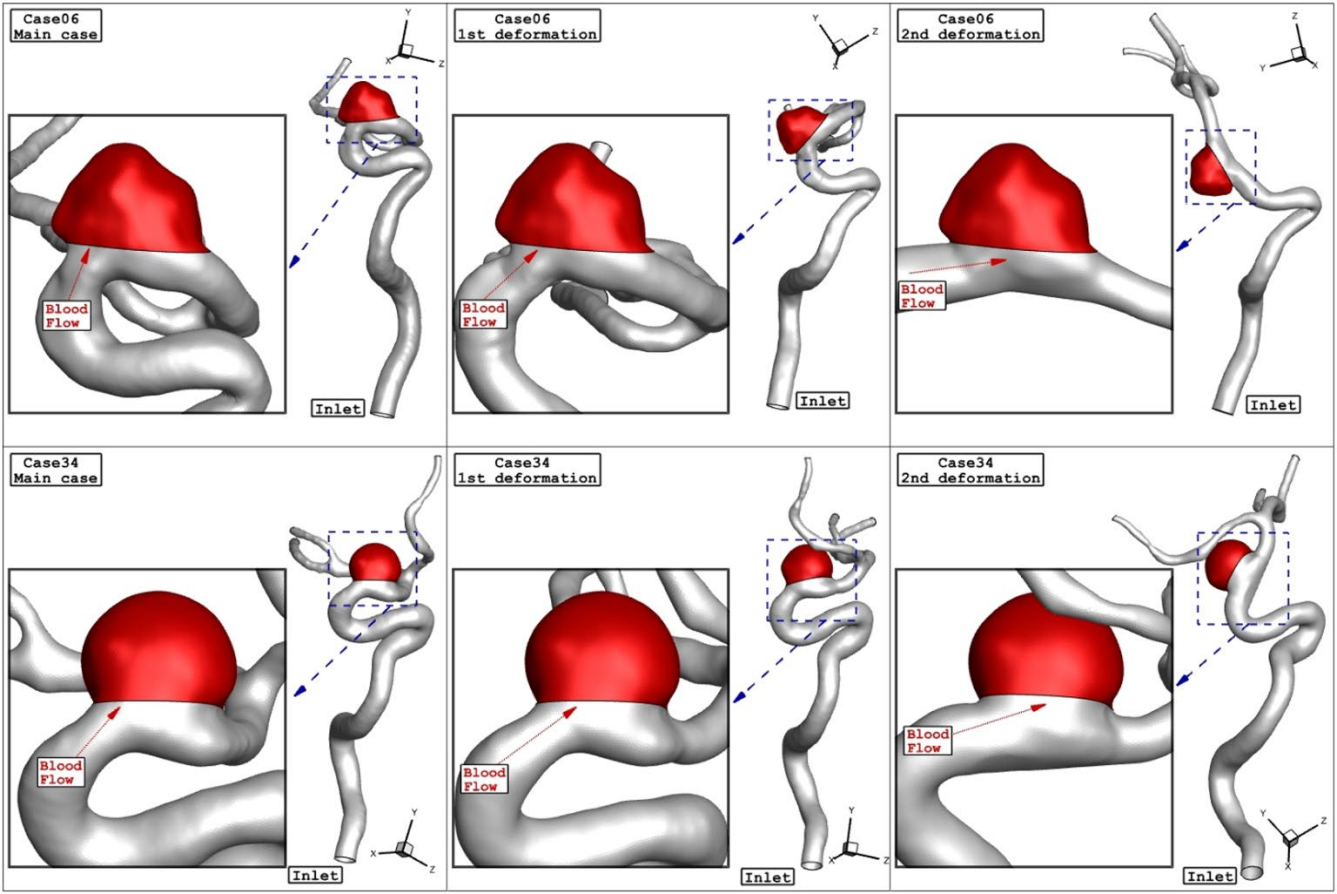
The geometry of cases 06 and 34 and their deformations.

**Figure 9:**
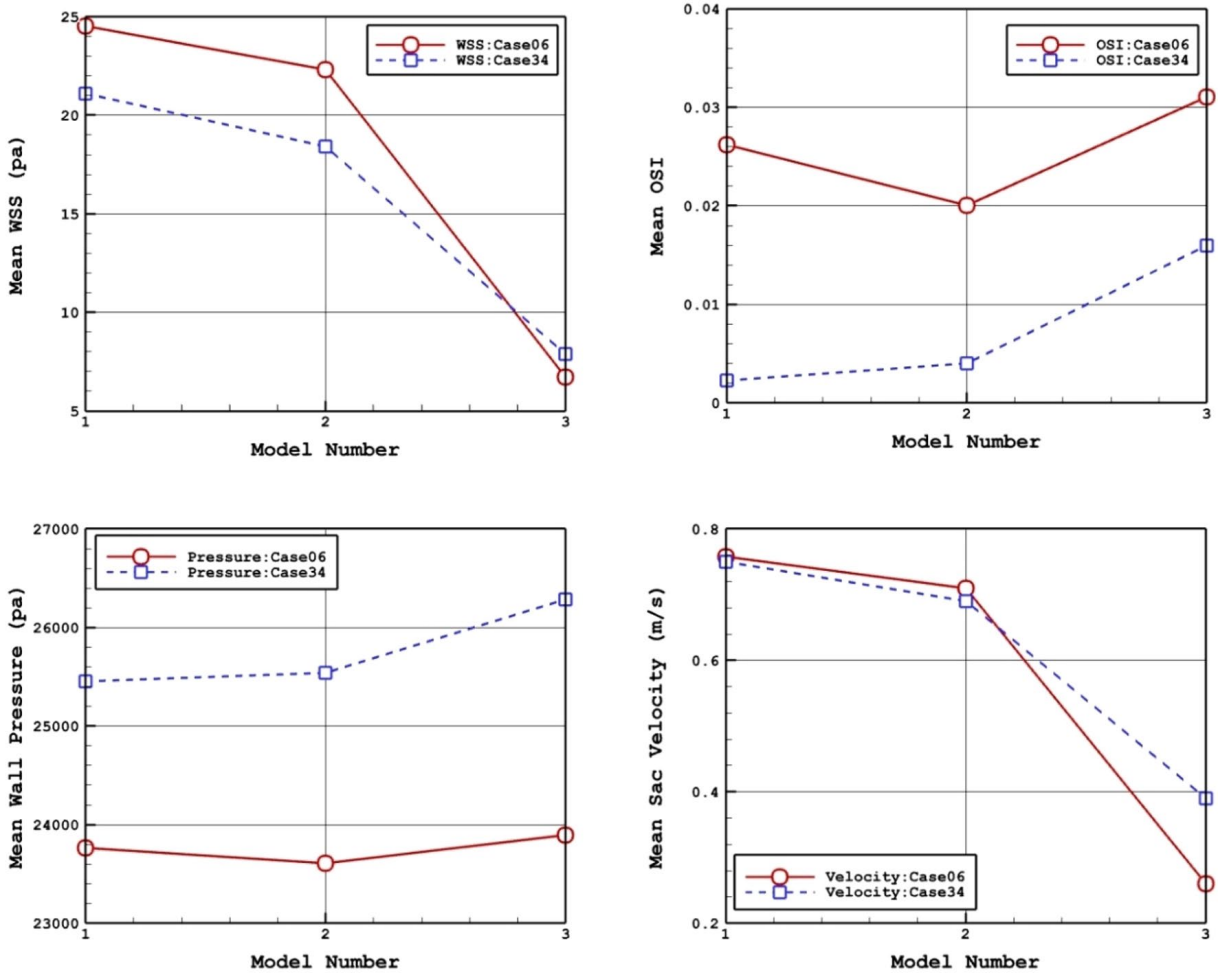
Deformation effects on mean values of WSS, OSI, sac wall pressure, and sac velocity.

As explained earlier, one of side effects of the stent is to change the angle of blood inflow with normal vector of ostium area. Figure 8 illustrates the 2 stages of transitional deformation process in which the orientation of the blood stream with ostium are changed. The hemodynamic characteristics of these two cases in these two deformation conditions are illustrated in Fig. 9. Comparison of the mean WSS and velocity indicates that these factors decreases about 8% and 72% at the 1st and 2nd deformations, respectively. The impacts of the deformation on the mean OSI and pressure value is not significant.

**Figure 10:**
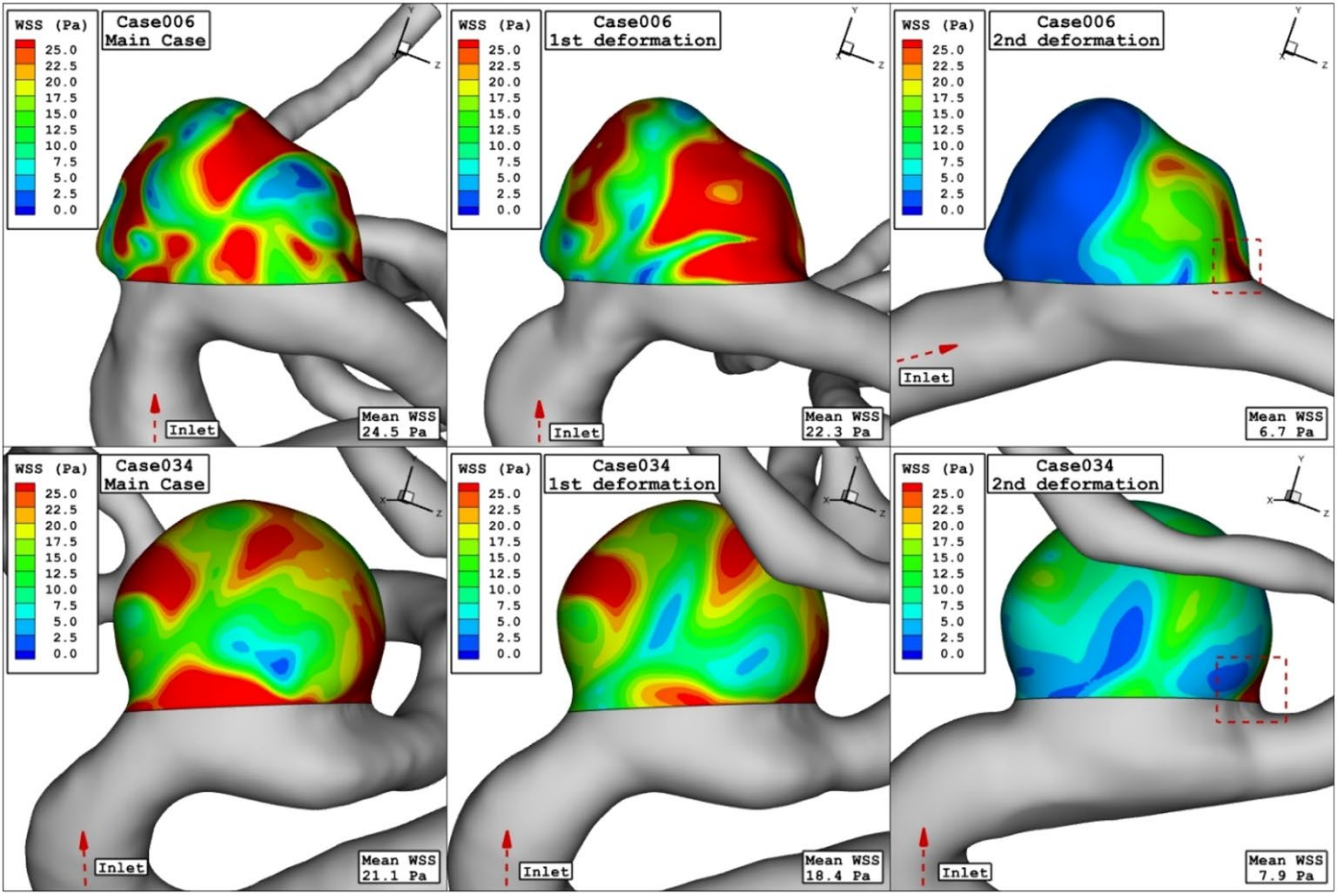
WSS contours (peak systolic) in different neck vessel angle.

**Figure 11:**
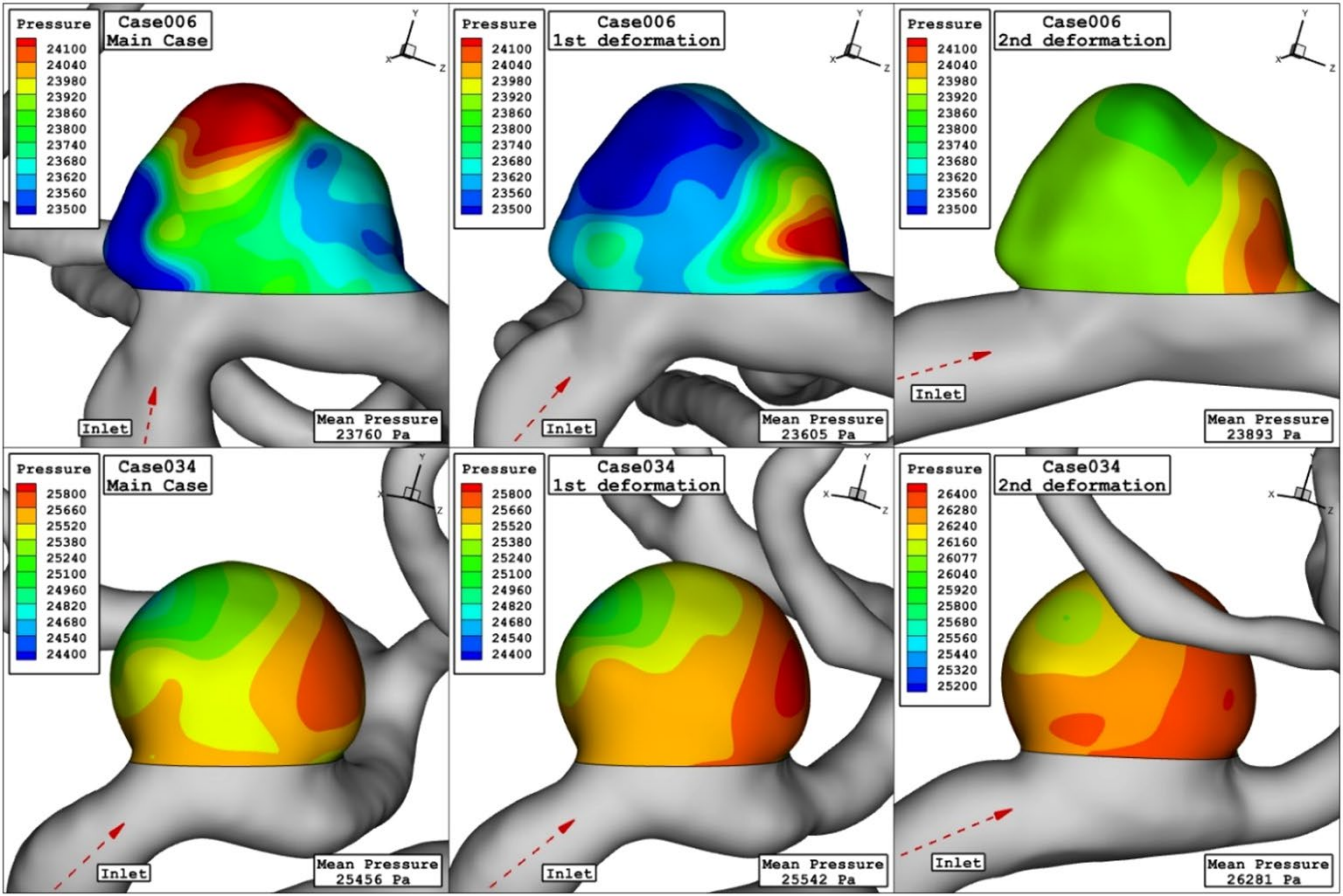
Wall pressure contours (Peak systolic) in different neck vessel angle.

**Figure 12:**
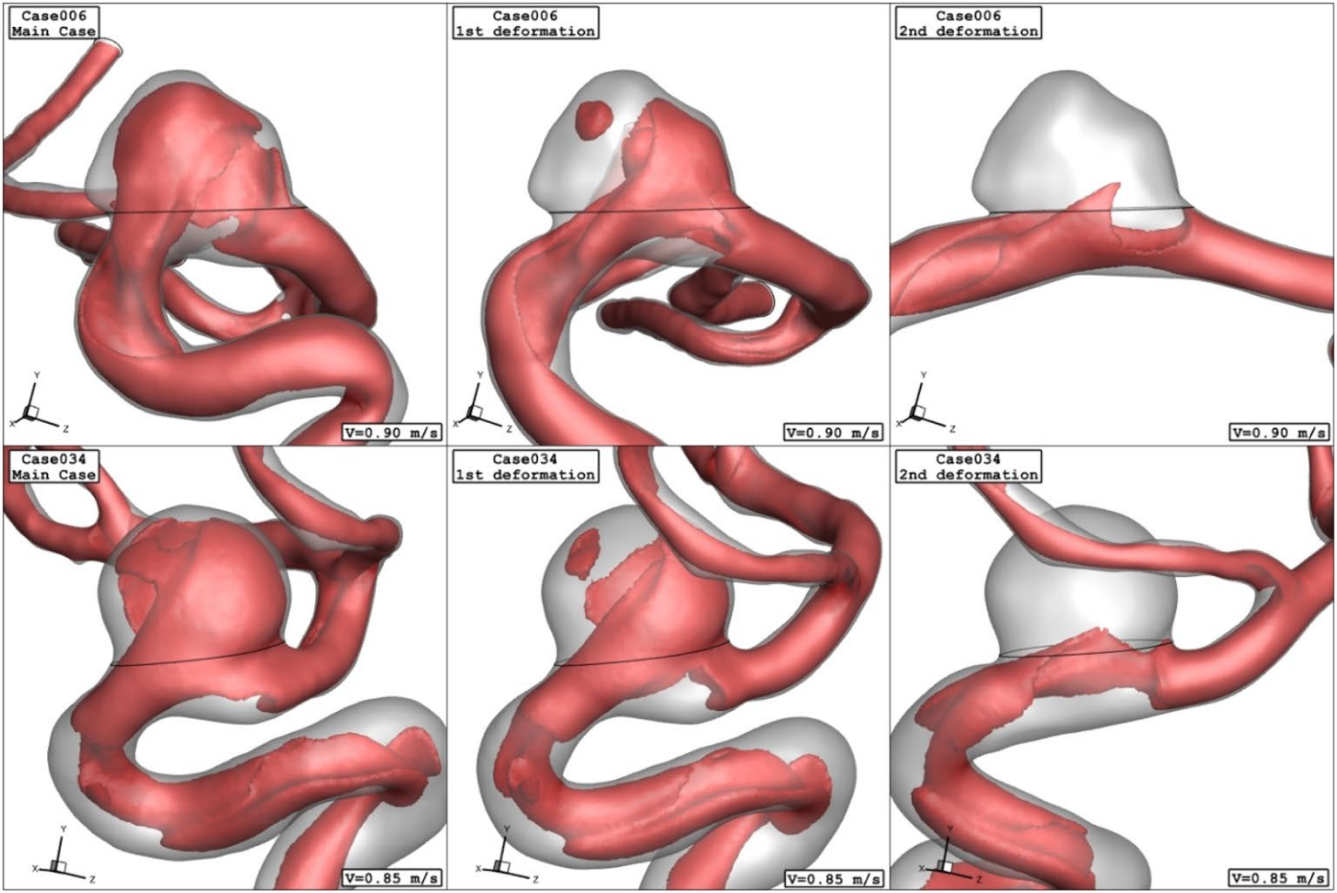
Iso-surface (velocity at peak systolic) in different neck vessel angle.

The impacts of the deformation are clearly observed on the distribution of the WSS as shown in Fig. 10. Since the orientation of the blood stream with sac ostium is changed in the deformation, limited blood stream enters into the sac and this significantly reduces the WSS on the sac wall. Besides, the tension of the blood stream on the aneurysm wall is less after 2nd deformation. The pressure contour (Fig. 11) also confirm that the location of the high-pressure region changes via the deformation and transfer from the dome section to neck region. In fact, the blood entrance into the aneurysm sac occurs in this section in the 2nd deformation stage. As it is observed, in the main cases, the blood flow bifurcates after first contact on the aneurysm wall and shapes the dome as noticed in the pressure distribution. The iso-surface blood velocity also disclosed the impact of deformation on the blood flow feature inside the sac (Fig. 12). It is observed that the blood stream tends to moves along the parent vessel in the 2nd deformation model and this is identical in both cases.

## Conclusion

In this work, computational fluid dynamic is used for the modeling of the blood stream inside cerebral ICA aneurysms. The impacts of endovascular coiling on blood hemodynamic are investigated by applying porosity inside the sac. Also, the deformation of the aneurysm by the effects of stent is also studied in two different stages. WSS, pressure and OSI value of selected aneurysm are compared in different stages of blood cycles. Obtained results indicates that the mean WSS is reduced up to 20% via coiling of the aneurysm while the deformation of the aneurysm (applying stent) could reduce the mean WSS up to 71%. In fact, the impacts of limiting blood stream by deformation is more than coiling on protection and control of aneurysm growth and probable rupture.

## Data Availability

All data generated or analysed during this study are included in this published article.
